# An epidemiological assessment of hemodialysis, continuous kidney replacement therapy, and peritoneal dialysis in pediatric acute kidney injury patients

**DOI:** 10.1080/0886022X.2026.2706951

**Published:** 2026-08-10

**Authors:** Sanat Subhash, Ethan Kalina, Jacob Almeda, Arwa Nada, Aadil Kakajiwala, Rupesh Raina

**Affiliations:** ^a^ Northeast Ohio Medical University, Rootstown, OH, USA; ^b^ Internal Medicine and Pediatrics, The MetroHealth System/Case Western Reserve University Residency Program, Cleveland, OH, USA; ^c^ Division of Pediatric Nephrology, Department of Pediatrics, Loma Linda University, Loma Linda, CA, USA; ^d^ Divisions of Pediatric Nephrology and Critical Care Medicine, Children’s National Hospital, Washington, DC, USA; ^e^ Akron Nephrology Associates/Cleveland Clinic Akron General Medical Center, Akron, OH, USA

**Keywords:** Pediatric acute kidney injury, kidney replacement therapy, continuous kidney replacement therapy, hemodialysis, Peritoneal dialysis, intensive care outcomes

## Abstract

**Introduction:**

Hemodialysis (HD), peritoneal dialysis (PD), and continuous kidney replacement therapy (CKRT) are major modalities of kidney replacement therapy (KRT) in pediatric acute kidney injury (AKI). This study uses TriNetX data to identify clinical characteristics of pediatric AKI patients receiving HD, PD, and CKRT and compare outcomes across 30-, 90-, 180-, and 365-day follow-ups.

**Methods:**

Pediatric patients aged 0–18 years with AKI (August 2004–August 2024) were identified using ICD-10 and CPT codes within the TriNetX U.S. Network. Three cohorts (HD, PD, CKRT) were analyzed for outcomes and comorbidities. Kidney transplant recipients were excluded. Cohorts were not propensity matched to reflect real-world disease burden. A total of 7,476 pediatric patients (mean age ∼9 years) initiated KRT [HD 39.1% (*n* = 2,929), PD 41.9% (*n* = 3,139), CKRT 18.8% (*n* = 1,408)].

**Results:**

CKRT patients had higher BUN (19.7 mg/dL) and glucose (136 mg/dL). Intestinal diseases were frequent comorbidities. Hypertension was most common in CKRT (12.1%). Diuretics (12.1%) and epinephrine (11.3%) were the most used medications. At 30 days, mortality was lowest in HD (13.7%) vs PD (14.9%) and CKRT (19.9%). This persisted at 365 days (CKRT 24.2%). Ventilation rates were 22.0% (HD), 23.1% (PD), 25.6% (CKRT). ICU admission was highest in CKRT (71.1%).

**Conclusions:**

CKRT had the greatest ten-year incidence (3.6%) and prevalence (3.8%). HD and PD had lower observed mortality and ICU admission than CKRT, a pattern likely reflecting confounding by indication rather than a causal effect of modality. CKRT patients showed higher comorbidity burden and mortality, emphasizing the need for individualized management in pediatric AKI.

## GRAPHICAL ABSTRACT

**Figure:**
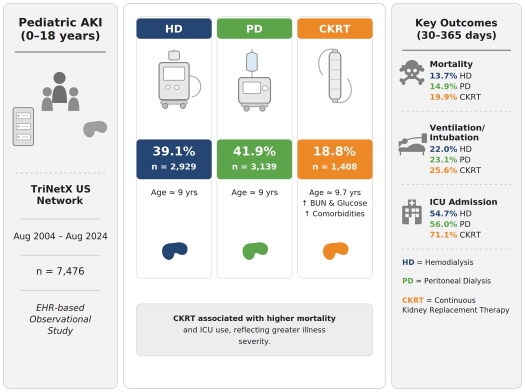

## Introduction

Acute Kidney Injury (AKI) is common among pediatric patients, affecting 10-35% of critically ill children and 34% of non-critically ill children [1]. Pediatric AKI is associated with higher risk of prolonged intensive care unit (ICU) stay, extended ventilation duration, and elevated mortality rates [1–9].

Hemodialysis (HD), peritoneal dialysis (PD), and continuous kidney replacement therapy (CKRT) are three major modalities of kidney replacement therapy (KRT) treatment for AKI in pediatric patients. Pediatric KRT has a prevalence of 100 per million children in the United States, with CKRT largely utilized within critically ill pediatric patients [10,11]. CKRT differs from HD due to its slower but prolonged clearance, with increased hemodynamic stability [12]. PD on the other hand helps with gradual solute clearance and ultrafiltration, offering lower overall clearance than CKRT. PD is commonly utilized in hemodynamically stable patients or in settings with limited vascular access for HD and CKRT [10]. Both pediatric HD, PD and CKRT report high rates of complications and associated mortality [13]. There is paucity of data on the incidence, prevalence of these modalities, and the demographic differences of the children receiving them across US pediatric populations [14,15]. Further research determining major comorbidities and contributors to HD, PD, and CKRT initiation can help improve acute and chronic pediatric AKI patient outcomes in the United States.

AKI, chronic kidney disease (CKD), and ESKD are major contributors to dialysis initiation in pediatric patients [16–19]. Frequently, comorbidities including cardiovascular, liver, and pulmonary diseases can negatively impact survival outcomes [20–24].

AKI in adults is often associated with chronic conditions such as advanced age, hypotension, atherosclerosis, while pediatric AKI is more frequently linked to acute conditions such as dehydration, sepsis, surgical procedures, and exposure to nephrotoxic medications. Recent international data analyzing treatment methodology for pediatric AKI suggest that modality choice is strongly influenced by patient hemodynamic stability, resource availability, and institutional expertise, with CKRT increasingly replacing PD as the preferred option in PICU settings due to precise volume control and treatment adaptability in unstable patients [25–28].

We hypothesized that children requiring CKRT represent a clinically more severe population, exhibiting higher comorbidity burdens and poorer outcomes compared with those receiving HD or PD.

We conducted a retrospective cohort analysis using electronic health record (EHR) data to identify clinical characteristics in HD, PD, and CKRT AKI pediatric patient cohorts and conduct a clinical outcomes analysis of treatment cohorts across 30, 90, 180, and 365-day follow-ups post-treatment.

## Methods

### Data searches and sources

We performed a retrospective cohort analysis through the TriNetX EHR database. TriNetX United States Network includes 69 Health-Care Organizations (HCOs) and more than 120 million patients.

The TriNetX database is de-identified following HIPAA requirements protecting patient privacy. Epidemiological and clinical data including patient demographics, diagnoses, procedures, laboratory values, and genomic information are provided for statistical analysis on the platform. Pediatric patients were selected in queries made through utilization of International Classification of Diseases, Tenth Revision (ICD-10) diagnosis codes and Current Procedural Terminology (CPT) procedure codes.

Study Design:

This study has two objectives:

1. To analyze the prevalence of major clinical characteristics and demographic variations in pediatric HD, PD, and CKRT patients to determine burden of disease.
2. To conduct a clinical outcomes analysis of HD, PD, and CKRT treatment, assessing variations in intensive care unit (ICU) admissions, mechanical intubation/ventilation, and all-cause mortality risk occurring after dialysis initiation, at 30, 90, 180, and 365-day follow-ups post-treatment.

### Participant selection and exclusion criteria

Pediatric (0–18 years of age) patients from August 2004 to August 2024 were included in 3 treatment cohorts – HD, PD and CKRT, created through ICD and CPT codes. A broad 2004–2024 timeframe was selected to capture longitudinal trends and ensure adequate sample size, recognizing that practice patterns have evolved but maintaining a uniform analytic approach across all years. AKI was defined as new-onset acute kidney injury during hospitalization, without preexisting chronic kidney disease. Kidney transplant status patients were excluded from this analysis. All patients were treated at hospitals across the United States, with the choice of PD as an initial modality for AKI in some centers reflecting institutional expertise and potential preference for less invasive approaches in smaller pediatric patients in settings lacking immediate CKRT or HD availability. Patients were categorized according to the first recorded dialysis modality to ensure mutually exclusive cohort assignment, and transitions between modalities were not separately analyzed.

Cohort 1, 2, and 3 were utilized for the comparative outcomes comorbidities analysis. Propensity matching was not performed to characterize real-world differences in patient acuity and management patterns across modalities rather than to infer causal relationships.

All cohorts were then used for a clinical outcomes comparison to determine areas of disparities and evaluate longitudinal treatment outcomes for HD, PD, and CKRT treatments across 30, 90, 180, and 365-day follow-up periods.

### Statistical analysis

All the three cohorts [HD, PD and CKRT] were assessed for demographic variables (age, sex, ethnicity, race), comorbidities (related to endocrine, nutritional and metabolic; circulatory system; genitourinary system; digestive system; musculoskeletal system and connective tissue), medications (epinephrine, norepinephrine, diuretics, vasopressin), and laboratory findings (sodium, potassium, chloride, bicarbonate, urea nitrogen, creatinine, albumin). The continuous data has been presented as mean (standard deviation) while categorical data as frequency (percentages). The measures of association identified individuals with specific outcomes [ICU readmission, mechanical intubation/ventilation, and all-cause mortality risk] occurring after dialysis initiation at 30, 90, 180, and 365-day intervals in all three cohorts. The epidemiology of HD, PD, or CKRT initiation in pediatric patients with AKI has been reported as incidence %, prevalence % and incidence rate (cases per 1 million person-days). Laboratory values represent aggregated measurements available within the TriNetX platform during the index hospitalization and are not standardized to peak AKI severity or dialysis initiation timing.

The descriptive analyses were performed using built-in analytics tools in the TriNetX platform. STROBE (The Strengthening the Reporting of Observational studies in Epidemiology) guidelines for cohort studies were followed for this investigation [29].

## Results

### Patient characteristics

From Table 1, the study included a total of 7,476 pediatric AKI patients who received KRT. The proportion treated with PD was the highest at 41.9% (*n* = 3139), followed by HD at 39.1% (*n* = 2929) and CKRT at 18.8% (*n* = 1408). Among these patients, 55.7% were male and 44.2% were female.

**Table 1.** Frequency distribution of characteristics by treatment cohort: HD, PD & CKRT.

Characteristic				
(Categorical variable)	*n*, %	HD *n*, %	PD *n*, %	CKRT *n*, %
Total	7476, 100	2929, 39.1	3139, 41.9	1408, 18.8
Sex				
Male	4171, 55.7	1625, 55.5	1754, 55.9	792, 56.3
Female	3305, 44.2	1304, 44.5	1385, 44.1	616, 43.7
Ethnicity				
Non-Hispanic	5021, 67.1	1986, 67.8	2117, 67.4	918, 65.2
Hispanic	1298, 17.3	507, 17.3	549, 17.5	242, 17.2
Unknown	1157, 15.4	436, 14.9	473, 15.1	248, 17.6
Race				
White	3733, 49.9	1464, 50	1569, 50	700, 49.7
African American	1979, 26.4	784, 26.8	830, 26.4	365, 25.9
Asian	299, 3.9	114, 3.9	122, 3.9	63, 4.5
Native Hawaiian or other Pacific Islander	90, 1.2	34, 1.2	35, 1.1	21, 1.5
Other	541, 7.2	212, 7.2	238, 7.6	91, 6.5
Unknown	803, 10.7	308, 10.5	332, 10.6	163, 11.6
Comorbidities				
Hypertension	692, 9.2	238, 8.1	283, 9	171, 12.1
Type 2 diabetes mellitus	157, 2.1	60, 2	67, 2.1	30, 2.1
Overweight, Obesity	408, 5.4	151, 5.2	167, 5.3	90, 6.4
Ischemic heart disease	65, 0.8	19, 0.6	26, 0.8	20, 1.4
Cerebrovascular disease	355, 4.7	124, 4.2	144, 4.6	87, 6.2
Stomach/duodenum disease	811, 10.8	307, 10.5	335, 10.7	169,12
Liver disease	448, 5.9	150, 5.1	185, 5.9	113, 8
Other intestinal diseases	1308, 17.4	494, 16.9	541, 17.2	273, 19.4
Tubulo-interstitial Diseases	202, 2.7	77, 2.6	86, 2.7	39, 2.8
Glomerular Disease	30, 0.4	10, 0.3	10, 0.3	10, 0.7
Other kidney/ureter disorder	305, 4.0	106, 3.6	131, 4.2	68, 4.8
Other urinary disease	507, 6.7	203, 6.9	214, 6.8	90, 6.4
Connective tissue disease	182, 2.4	69, 2.4	78, 2.5	35, 2.5
Mood disorders	374, 5.0	145, 5	152, 4.8	77, 5.5
Medications				
Epinephrine	852, 11.3	320, 10.9	353, 11.2	179, 12.7
Norepinephrine	210, 2.8	72, 2.5	84, 2.7	54, 3.8
Vasopressin (USP)	74, 0.9	26, 0.9	29, 0.9	19, 1.3
Diuretics	909, 12.1	334, 11.4	380, 12.1	195, 13.8

Non-Hispanic/Non-Latino children constituted 67.1% of the cohort, while 50% were White, followed by Black children at 26.4%. Comorbidity profiles were generally comparable across groups, with hypertension (8–12%), stomach and duodenal diseases (10–12%), other intestinal diseases (16–19%), and liver disease (5–8%) being the most common. The CKRT cohort had higher proportions of comorbidities compared with the other cohorts, with the highest proportion being other intestinal diseases at 19.4%, followed by hypertension at 12.1%, stomach and duodenal diseases at 12.0%, liver disease at 8.0%, cerebrovascular disease at 6.2%, and glomerular disease at 0.7%. Medication exposure varied by modality. Diuretics at 12.1% and epinephrine at 11.3% were the most frequently used medications across all treatment cohorts, followed by norepinephrine at 2.8% and vasopressin at 0.9%.

From Table 2, Although the mean age at index/event was approximately 9 years across all cohorts, the CKRT cohort had a slightly higher mean age of 9.7 years. Complete baseline laboratory and medication data were also available in Table 2 for all included pediatric patients. Mean sodium level was 139 mEq/L and mean creatinine was 0.79 mg/dL overall. Mean Blood Urea Nitrogen was higher in CKRT patients at 19.7 mg/dL compared to HD and PD at 15.4 mg/dL and 16.4 mg/dL, respectively, reflecting a greater metabolic burden at the time of initiation. Mean potassium levels were similar across modalities at approximately 4.2 to 4.3 mmol/L, indicating comparable electrolyte profiles at the time of therapy initiation.

**Table 2.** Mean distribution of characteristics by treatment cohort: HD, PD & CKRT.

Characteristic (continuous variable)	HD µ, SD	PD µ, SD	CKRT µ, SD
Age (years)			
Age at index	9.1, 5.5	9.0, 5.5	9.7, 5.0
Current Age	12.6, 4.9	12.6, 4.9	13.4, 4.4
Laboratory findings			
Sodium (mEq/L)	139, 4.6	139, 4.7	140, 5.6
Potassium (mEq/L)	4.2, 0.7	4.3, 0.7	4.3, 0.7
Bicarbonate (mEq/L)	23.6, 4.2	23.5, 4.3	23.1, 4.6
Chloride (mEq/L)	105, 5.7	105, 5.7	105, 6.1
Blood urea nitrogen (mg/dL)	15.4, 12.3	16.4, 14.0	19.7, 18.9
Creatinine (mg/dL)	0.79, 4.9	0.80, 4.8	0.77, 0.7
Glucose (mg/dL)	123, 71.5	124, 71.2	136, 89.3

### Clinical outcomes

**Figure 1.**
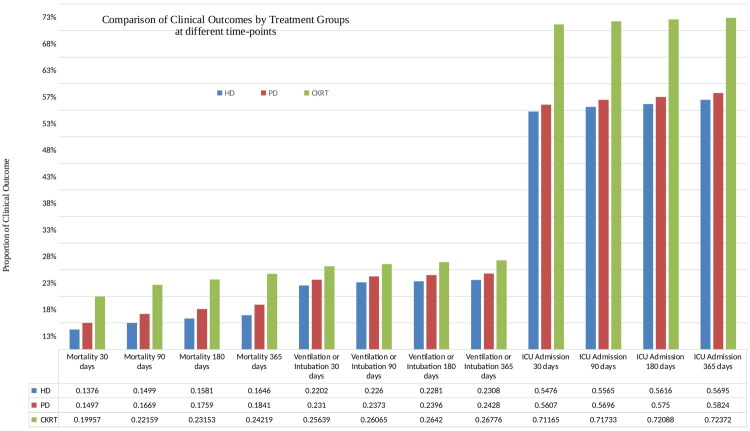
Comparison of clinical outcomes by treatment groups. 30-, 90-, 180-, and 365-day outcomes for all-cause mortality, ventilation/intubation, and ICU admission across the three modalities.

Table 3 and Figure 1 show the proportion of pediatric patients with all-cause mortality, ICU admission and ventilation/intubation across the three cohorts at different time-points.

**Table 3.** Comparison of clinical outcomes by treatment groups at different time points.

Clinical outcome	Time-point (days)	HD %	PD %	CKRT %
All-cause mortality	30	13.7	14.9	19.9
	90	14.9	16.6	22.1
	180	15.8	17.5	23.1
	365	16.4	18.4	24.2
Ventilation or intubation	30	22.0	23.1	25.6
	90	22.6	23.7	26.0
	180	22.8	23.9	26.4
	365	23.0	24.2	26.7
ICU admission	30	54.7	56.0	71.1
	90	55.6	56.9	71.7
	180	56.1	57.5	72.0
	365	56.9	58.2	72.3

ICU admission and mechanical ventilation were evaluated as post-initiation outcomes and were not used as baseline severity indicators at the time of dialysis initiation.

All-cause mortality at 30 days post-KRT initiation was highest in CKRT at 19.9%, followed by PD at 14.9% and HD at 13.7%. At 90 days, mortality increased to 22.1% in CKRT, 16.6% in PD, and 14.9% in HD. At 180 days, CKRT mortality was 23.1%, compared with 17.5% in PD and 15.8% in HD. By 365 days, all-cause mortality was 24.2% in CKRT, 18.4% in PD, and 16.4% in HD.

The proportion of patients with ventilation or intubation at 30 days was lower in HD group at 22% and PD at 23.1% as compared to CKRT group at 25.6%. By 90 days, ventilation rates rose to 26.0% in CKRT, 23.7% in PD, and 22.6% in HD. At 180 days, rates were 26.4% in CKRT, 23.9% in PD, and 22.8% in HD, increasing slightly by 365 days to 26.7% in CKRT, 24.2% in PD, and 23.0% in HD.

The proportion of patients with ICU admission at 30 days was lower in HD at 54.7% and PD at 56.0% as compared to CKRT at 71.1%. ICU admission increased to 71.7% in CKRT, 56.9% in PD, and 55.6% in HD at 90 days. At 180 days, rates were 72.0% in CKRT, 57.5% in PD, and 56.1% in HD, and 72.3% in CKRT, 58.2% in PD, and 56.9% in HD at 365 days.

Across all time points, clinical outcome rates remained highest in the CKRT cohort, followed by PD and HD. No statistical tests were performed; outcomes are reported descriptively.

## Ten-year epidemiology of KRT modality use in pediatric AKI

The 10-year incidence and prevalence of HD, PD and CKRT initiation in pediatric patients with AKI is reported in Table 4. Incidence over the study period was highest for CKRT (3.6%), compared to HD (1.1%) and PD (0.2%). Prevalence followed a similar pattern, with CKRT (3.8%), HD (1.1%), and PD (0.2%) (Table 4).

**Table 4.** Ten-Year Incidence and prevalence proportions of HD, PD and CKRT treatments.

Group	HD			PD			CKRT		
	Inc %	Pre %	IR	Inc %	Pre %	IR	Inc %	Pre %	IR
Age									
All ages	1.1	1.1	7	0.2	0.2	1	3.6	3.8	22
0–4 years	1.2	1.3	7	0.1	0.1	0	3.8	4.1	22
5–9 years	0.9	1.0	6	0.2	0.2	2	3.4	3.6	21
Sex									
Female	1.2	1.3	8	0.1	0.1	0	3.5	3.7	21
Male	0.9	0.9	6	0.2	0.2	2	3.7	4.0	22
Race									
White	1.2	1.2	7	0.1	0.1	0	3.7	4.0	21
African American	1.0	1.0	5	0.5	0.5	3	3.3	3.4	18
Asian	2.2	2.2	15	0.0	0.0	0	3.0	3.2	20
Unknown	0.7	0.7	6	0.6	0.6	5	3.1	3.2	25
Other	1.5	1.5	10	0.0	0.0	0	5.2	5.4	35
Ethnicity									
Hispanic	0.9	0.9	5	0.0	0.0	0	4.0	4.3	23
Non-Hispanic	1.1	1.2	7	0.2	0.2	1	3.5	3.7	20
Unknown	1.1	1.1	9	0.5	0.5	4	3.6	3.7	29

Inc-Incidence proportion, Pre- prevalence proportion; IR - Incidence Rate (Number of cases/million person-days).

Incidence rates per million person-days were 22.0 in CKRT, 7.0 in HD, and 1.0 in PD. Within the CKRT cohort, males had a slightly higher prevalence (4.0%) than females (3.7%). Among racial subgroups, Asian children receiving HD had the highest incidence proportion (2.2%), while Black children had lower HD incidence (1.0%) but relatively higher PD use (0.5%).

CKRT reported an incidence rate of 22.0 cases per million person-days. In children of all ages, CKRT incidence was increased. PD consistently showed the lowest utilization across all age and demographic subgroups, with an overall incidence of 0.2%. Among patients with unknown ethnicity, CKRT incidence and prevalence were 3.6 and 3.7%, respectively.

## Demographic stratification of modality use

The incidence and prevalence were also stratified based on different demographic attributes. CKRT prevalence was highest among children aged 0–4 years (4.1%) and Hispanic patients (4.3%), whereas HD prevalence was more evenly distributed across sex and race (1.2% White, 1.0% Black). PD use was highest in males (0.2%) and Black children (0.5%), suggesting underlying demographic patterns in modality selection or access.

## Discussion

We performed a retrospective cohort analysis utilizing the TriNetX EHR database to compare PD, HD and CKRT treatment for pediatric AKI patients and compare patient characteristics and longitudinal clinical outcomes between each modality.

All-cause mortality, ICU admission, and ventilation/intubation rates were assessed between all three cohorts at 30-, 90-, 180-, and 365-day time intervals and observed to be lower in the HD cohort. The PD cohort also demonstrated lower rates than CKRT, though slightly higher than HD, and CKRT reported higher proportions of comorbidities and more severe clinical outcomes. Additionally, various clinical characteristics including liver disease and hypertensive disorders were assessed to better understand their influence on patient health and treatment results. Our epidemiological findings provide the largest multi-institutional dataset to date analyzing modality-specific trends in pediatric AKI, revealing distinct patterns of patient selection and outcome disparities. These comparisons are descriptive and unadjusted, and the observed differences across modalities are most consistent with confounding by indication, in which baseline illness severity and hemodynamic instability drive modality selection, rather than with a causal effect of the modality itself.

The HD cohort showed numerically lower mortality rates than CKRT, with 13.7% at 30 days and 16.4% at 365 days, whereas CKRT showed 19.9% at 30 days and 24.2% at 365 days. The PD cohort had intermediate mortality rates, 14.9% at 30 days and 18.4% at 365 days. Treatment modality is of utmost importance in treatment of AKI, and the lower mortality rates in the HD and PD cohort compared to CKRT highlight that importance. Mortality is a heavily studied outcome pertaining to pediatric AKI, with multiple studies indicating increasing mortality rates directly correlated to AKI staging [30–33]. *Banigan* et al. conducted a retrospective cohort study showing that longer times to CKRT initiation were associated with higher odds of mortality [34].

These findings help add to the existing literature on the severe outcomes in pediatric populations when deciding CKRT, PD or HD treatment, including ICU admissions. The difference in mortality observed between CKRT and HD/PD reflects illness severity but may also indicate delayed escalation to CKRT in rapidly deteriorating patients, emphasizing the importance of timely recognition and intervention in AKI progression.

ICU admissions were numerically lower in the HD cohort in comparison to the CKRT cohort at both 30- and 365-day intervals (54.7 and 56.9%, respectively), with the PD cohort showing similar results (56.0 and 58.2%). The lower ICU admission rate for HD and PD patients likely reflects the severity of illness, or lack thereof, compared to those receiving CKRT. This finding reflects the current literature, as CKRT is the mainstay of treatment for pediatric AKI ICU admits while HD and PD patients are consistently less sick [34]. Importantly, CKRT is a more controlled method of fluid and solute removal in comparison to HD. Slow correction of renal failure allows providers to avoid acute side effects of traditional dialysis, including rapid fluid and electrolyte shifts. In our cohort, CKRT patients represented the most critically ill subgroup, consistent with prior findings that CKRT is preferentially used in unstable patients [35,36].

Recognition of hemodynamic instability is critical, allowing providers to quickly initiate treatment to avoid long term deficits. From existing literature and our data, quick recognition of hemodynamic instability likely means quick preparation for CKRT. Hemodynamically unstable patients face higher rates of mortality and prolonged ICU admissions when there is a delay in the initiation of CKRT [30,34,37]. Other studies have found direct relationships between time to CKRT initiation and survival time after pediatric ICU admission, demonstrating the importance of early recognition of AKI in the hospital setting [37,38].

In a large retrospective cohort of 190 children receiving CKRT, nonsurvivors had a longer median time to initiation compared with survivors (3.4 vs 2.0 days, *p* = 0.001), and delay in initiation independently increased mortality risk (adjusted OR 1.05; 95% CI, 1.01–1.11) [37,38].

When patients are showing signs of renal recovery post-CKRT treatment, there is limited research on what criteria define recovery and what factors meaningfully impact time to recovery. Commonly, creatinine is used to analyze successful treatment.

In our cohort, measured serum creatinine values were similar across modalities (CKRT 0.77 mg/dL, HD 0.79 mg/dL, PD 0.80 mg/dL). These values represent laboratory measurements obtained around the index dialysis period rather than standardized pre-initiation or peak values, and were not standardized for timing of measurement, age, muscle mass, or fluid status. They are therefore reported descriptively and should not be interpreted as indicators of renal recovery or comparative treatment efficacy. Schiffl et al. found that the best predictor of recovery in patients showing signs of excretory renal function is a 24-h creatinine clearance of at least 20 mL/min with decreasing serum creatinine levels during that same time period [39]. Increasing research on pediatric renal failure recovery will help physicians recognize and more effectively treat pediatric AKI patients.

Ventilation and intubation rates were also higher in the CKRT cohort compared to the HD and PD cohorts at all intervals. At 30 days, CKRT showed a rate of 25.6%, compared to 22.0% in HD and 23.1% in PD. An increase in mechanical ventilation and intubation in CKRT patients is expected, coinciding with our data showing pediatric ICU admission rates. *Uber* et al. conducted a systematic review of 59 studies showing pediatric AKI patients have an increased predisposition to mechanical ventilation and intubation, emphasizing the complexity of treating pediatric AKI patients presenting with a wide variety of conditions [30].

Hypertensive disease was less prevalent in the HD cohort (8.1%) when compared to CKRT (12.1%) and PD (9.0%). The higher comparative prevalence of hypertension to other comorbidities in our study aligns and reinforces with findings by *Patel* et al. who noted that hypertension was associated with 19% of pediatric AKI patients [40].

Liver disease was more prevalent in the CKRT cohort (8%) compared to HD (5.1%) and PD (5.9%). Disease of the liver, when combined with AKI, is associated with higher odds or morbidity and mortality, reflecting the importance of CKRT for patients who are more critically ill than HD or PD eligible patients. Liver disease is a common comorbidity in pediatric AKI patients due to increased stress on the kidneys [41].

In the HD and PD cohorts, recorded exposure to epinephrine (HD 10.9%, PD 11.2%), norepinephrine (HD 2.5%, PD 2.7%), and diuretics (HD 11.4%, PD 12.1%) was lower than in the CKRT cohort (epinephrine 12.7%, norepinephrine 3.8%, diuretics 13.8%). These medications were captured as binary EHR-derived indicators without timing, dose, duration, or indication, and are reported as observed proportions rather than as measures of shock burden or fluid-management strategy. This is consistent with the WE-ROCK study, a secondary analysis of renal replacement outcomes across 34 centers internationally, concluding that two-thirds of children and young adults who received CKRT needed vasopressor therapy [42,43]. This observation of greater instability in the CKRT cohort highlights the importance of fluid management, particularly to avoid fluid overload, a known factor in worsening AKI outcomes and complications in treatment.

In our analysis, PD reported the lowest incidence (0.2%) and prevalence (0.2%) compared to HD (1.1% and 1.1%) and CKRT (3.6% and 3.8%), consistent with trends reported in current literature.

While PD has historically been favored for pediatric AKI due to its feasibility, particularly in neonates, evolving practices and increasing availability of extracorporeal modalities such as CKRT and HD have led to a decline in PD use [10,44]. One European study reported that only 14% of pediatric AKI patients received PD, with the majority (64%) receiving CKRT [45]. Similarly, a survey of Taiwanese hospitals by Tain et al. comparing the three modalities found that the least used method of treatment was PD [46].

Our cohort findings reflect broader trends in pediatric AKI management, where modality selection is shaped by patient condition, institutional capacity, and clinician expertise [25,26].

Access caliber and modality selection varied based on patient size, hemodynamic status, and acuity level, with temporary central venous catheters commonly placed for both HD and CKRT across centers. Regional citrate anticoagulation was employed in the majority of CKRT cases, reflecting contemporary U.S. practice patterns aimed at maintaining circuit patency. In this cohort, CKRT patients exhibited the highest mortality at all intervals, consistent with prior studies associating CKRT use to poorer outcomes among patients with greater hemodynamic instability and vasopressor dependence [28].

The CKRT cohort also demonstrated the highest rates of ICU admission (71.1%) and mechanical ventilation or intubation (25.6%) at 30 days, aligning with previously identified mortality predictors such as vasopressor use and respiratory failure in critically ill children with AKI [27]. In contrast, the HD and PD cohorts showed lower rates of ICU admission (54.7%, 56.0%) and mechanical ventilation (22.0%, 23.1%), suggesting that HD and PD are more frequently used in relatively stable patients with fewer systemic complications. Comparable studies in broader AKI populations have shown that PD provides favorable quality-of-life outcomes and economic cost savings compared to HD, supporting potential quality improvement opportunities in treating select pediatric cases when hemodynamic stability allows [47,48]. These patterns support a risk-adapted treatment strategy that considers baseline clinical severity when selecting dialysis modality in pediatric AKI. The persistence of elevated ICU and ventilation rates in CKRT patients through one year suggests that the impact of modality selection extends beyond the acute phase and may influence long-term morbidity.

Modality selection also shapes supportive care needs in CKRT-treated patients who experience higher rates of hemodynamic instability, ICU admission, and mortality [49]. Critically ill children receiving CKRT are at increased risk for protein-energy wasting due to heightened catabolic stress and significant nutrient losses and require individualized nutrition approaches, such as indirect calorimetry and micronutrient supplementation [49].

In contrast, HD and PD patients are often more stable, allowing for earlier initiation of nutrition support with fewer metabolic complications [50]. Additionally, urine output greater than 0.5 mL/kg/hr within 6 h before discontinuation has been identified as a reliable predictor for successful CRRT liberation in pediatric patients [51]. When analyzing acute and chronic outcomes, CKRT patients may require more tailored nutritional and liberation strategies that may not be as critical in HD and PD cohorts.

Peritoneal dialysis (PD) remains a frontline treatment for neonates and infants due to the challenges in establishing vascular access for dialysis in this population. Compared to HD and CKRT, PD remains a more accessible, less invasive option and is associated with improved outcomes when initiated early in critically ill neonates [43]. This is especially useful for neonates who present post cardiovascular surgery, with literature noting that quicker initiation of PD in this patient population is directly correlated with better outcomes [40,44].

Our study also analyzed the difference in outcomes after stratification of cohorts by age, sex, and race. 50% of the data in each comparative outcomes cohort were collected from white children, while just under 20% for each in black children. Current research shows black children experience higher rates of AKI when compared to other races, most likely associated with a lower socioeconomic status [45–47].

Our data showed that Black children had relatively higher use of peritoneal dialysis but lower use of hemodialysis and continuous kidney replacement therapy compared with other groups, which may reflect systemic barriers to accessing resource-intensive modalities, delayed referral, or underutilization of intensive therapies.

Our findings highlight systemic barriers to nephrology care and underscore the need for equity-focused research to ensure timely, appropriate AKI interventions for diverse pediatric populations, particularly Black and Hispanic children.

Diabetes, a commonly studied and known risk factor for the development of AKI, was an even comorbidity between our three cohorts (2%). Diabetes causes chronic stress on the kidneys, marked by persistent albuminuria, hypertension, and a decreasing GFR at each visit [48]. Notably, treating AKI patients with diabetes should be categorized into two types of diabetes.

Patients with chronic diabetes-related damage are more prone to severe outcomes when compared to those with newly diagnosed Type 1 diabetes [52]. Conversely, diabetic patients can present with more acute onset problems rather than chronic causes. For instance, DKA is a common (acute) presentation for diabetic children. When type 1 diabetic patients present to the hospital in DKA, rates of AKI incidence vary from 41.5–56% [52–55].

Future prospective studies should explore whether targeted clinical pathways and early warning systems could reduce the time to CKRT initiation and potentially improve survival among high-risk pediatric subgroups. Obesity is associated with various hypermetabolic syndromes, increasing stress on the kidneys and predisposing pediatric patients to AKI [35,56,57]. The link between obesity, pediatric hypertension, and diabetes further increases the risk of AKI [58]. Specifically, investigations should focus on identifying optimal timing thresholds for CKRT initiation, evaluating modality transitions over the disease course, and incorporating granular physiologic data (e.g. fluid overload metrics, vasopressor dose, and hemodynamic indices) to refine risk stratification. Long-term follow-up is needed to assess renal recovery trajectories, progression to CKD, neurodevelopmental outcomes, and health-related quality of life across modalities. Additionally, equity-focused analyses examining racial, socioeconomic, and institutional disparities in access to HD, PD, and CKRT are warranted, alongside implementation studies testing early warning systems and standardized care pathways to reduce delays in therapy. Integrating nutritional, pharmacologic, and biomarker-driven approaches may further optimize individualized treatment strategies and improve survival and morbidity in this high-risk population.

## Limitations

This retrospective cohort analysis utilized data from the TriNetX database, which carries inherent limitations related to statistical and clinical variability. The TriNetX platform aggregates de-identified real-world EHR data across a federated network and has been used in prior clinical and epidemiologic research [59–61]. Due to the de-identified nature of the data, comparisons across hospital systems or care pathways were not possible. Additionally, reliance on EHRs introduces potential inaccuracies in patient conditions and outcomes, which may affect the validity of statistical tests. ICD and CPT coding limitations may also lead to underreporting or misclassification of conditions.

While TriNetX employs advanced data profiling and validation techniques to enhance data completeness, the analysis is restricted to 69 healthcare organizations, potentially excluding care received elsewhere. Longitudinal follow-up beyond one year was not included, limiting insights into long-term renal recovery and CKD progression.

Moreover, the observational nature of the data precludes causal inference, with findings reflecting correlations rather than definitive cause-effect relationships. Practice changes over two decades may have influenced modality selection and treatment protocols, but TriNetX data aggregation minimizes institutional bias. Although ICD-10 coding became standard in 2015, TriNetX harmonizes earlier ICD-9-CM data using the General Equivalence Mappings and internal curation processes, enabling consistent diagnostic classification across the study period. Medication exposure variables were limited to presence or absence within the EHR and lacked temporal alignment with dialysis initiation or indication, precluding granular assessment of vasopressor dependence or diuretic responsiveness.

Future analyses could further include nephrotoxic medication exposure and clinician decision-making variables, which may further clarify risk factors for AKI severity and RRT initiation. Despite these constraints, the study’s large sample size and strict exclusion criteria strengthen the comparative analysis between pediatric HD and CKRT cohorts. The predominance of white patients reflects regional and institutional demographics within TriNetX, underscoring the need for broader inclusion of racial and socioeconomic diversity in future AKI research to support equitable care and targeted interventions.

## Conclusion

Our retrospective cohort analysis revealed differences in clinical outcomes and characteristics among pediatric AKI patients treated with HD, PD, and CKRT. HD patients had the lowest rates of all-cause mortality, ICU admission, and ventilation/intubation, likely reflecting its use in more hemodynamically stable populations. PD patients also showed better outcomes than CKRT patients, though slightly less favorable than HD.

CKRT patients exhibited higher rates of hypertensive disease, liver disease, and glomerular disorders compared to HD patients. Diabetes and obesity rates were similar across all cohorts. Consistent with existing literature, our demographic analysis showed disproportionately high AKI rates among Black children.

Because treatment assignment in pediatric AKI is guided by baseline illness severity, these findings represent descriptive associations subject to confounding by indication and highlight the importance of individualized dialysis modality selection based on patient risk profiles. Tailored treatment strategies and early risk stratification can help reduce disparities and improve outcomes in pediatric AKI care. This large-scale descriptive study supports existing evidence and lays the groundwork for future clinical trials comparing real-world effectiveness and long-term outcomes across dialysis modalities.

## Data Availability

The data that support the findings of this study are available from TriNetX. Restrictions apply to the availability of these data, which were used under license for this study. Data are available https://live.trinetx.com with the permission of TriNetX.

## Clinical trial registration

No clinical trial registration was conducted as anonymized electronic healthcare record data was utilized for this study.

## Permission to reproduce material from other sources

No permission is required.
